# Etymologia: Rifampin

**DOI:** 10.3201/eid2403.ET2403

**Published:** 2018-03

**Authors:** Ronnie Henry

**Keywords:** rifampin, rifampicin, rifamycin, drugs, Rififi, tuberculosis, Piero Sensi

## Rifampin [rif-amʹpin]

In 1957, Piero Sensi and colleagues isolated a new bacterium, *Streptomyces mediterranei* (now *Amycolatopsis rifamycinica*) (Figure), from a soil sample from a pine forest in France. Material extracted from fermentation broths of *A. rifamycinica* contained microbiologically active substances that, as a group, were nicknamed Rififi. *Rififi* (French slang for “trouble”) was a 1955 French gangster film that was popular at the time and became the root of the name “rifamycin” for this group of antimicrobial agents. (Similarly, matamycin was originally nicknamed Mata Hari.) Rifampin (also known as rifampicin) is the *N*-amino-*N*ʹ-methylpiperazine (AMP) derivative of rifamycin.

**Figure:**
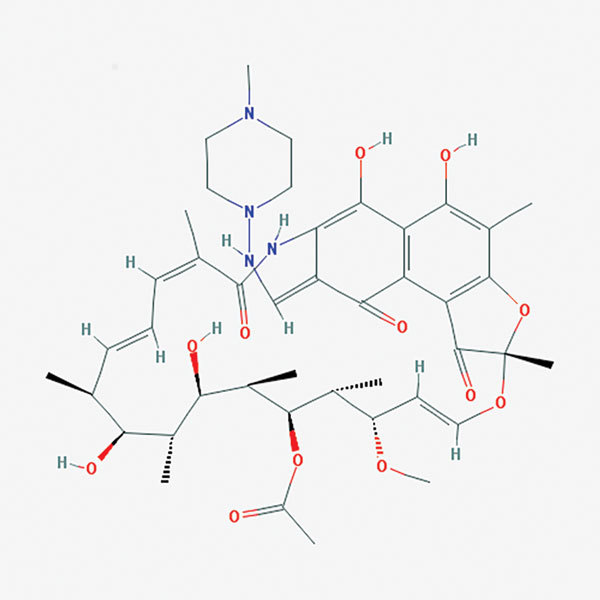
Chemical structure of Rifampin. Data deposited in or computed by PubChem, source: PubChem, URL: https://pubchem.ncbi.nlm.nih.gov
